# Forecasting of tilapia (*Oreochromis niloticus*) production in Bangladesh using ARIMA model

**DOI:** 10.1016/j.heliyon.2024.e27111

**Published:** 2024-02-24

**Authors:** Mohammad Abu Baker Siddique, Balaram Mahalder, Mohammad Mahfujul Haque, Mobin Hossain Shohan, Jatish Chandra Biswas, Shahrina Akhtar, A. K. Shakur Ahammad

**Affiliations:** a Department of Fisheries Biology and Genetics, Faculty of Fisheries, Bangladesh Agricultural University, Mymensingh, Bangladesh; b Department of Aquaculture, Faculty of Fisheries, Bangladesh Agricultural University, Mymensingh, Bangladesh; c Krishi Gobeshona Foundation, BARC Complex, Farmgate, Dhaka, Bangladesh

**Keywords:** Modeling, ARIMA, Auto regression, Moving average, Autocorrelation function, Estimating parameters, National fish policy, Tilapia production, Bangladesh

## Abstract

Tilapia farming has expanded rapidly in Bangladesh over the years thanks to a suitable climate for aquaculture and a consistently increasing demand for the fish rich in vitamins and minerals. A clear picture regarding the future trend of tilapia production in Bangladesh is still not available, however. The purpose of this study was to estimate parameters that fit into the Autoregressive Integrated Moving Average (ARIMA) model for forecasting tilapia production in Bangladesh. This was accomplished by calibrating and validating the ARIMA model taking into account the lowest values of the Akaike Information Criterion (AIC) and Bayesian Information Criteria (BIC), graphical arrangements of autocorrelation function (ACF) and partial autocorrelation function (PACF) plots. The best model derived was ARIMA (1, 1, 1), which showed an upward trend of tilapia production since 2006 to date and predicted a similar trend until the year 2040. If this trend continues, the yearly tilapia outturn in the country may reach 690,000 MT, with an upper limit of 1.15 million MT and lower limit of 0.23 million MT, reflecting a substantial increase of around 118% over that produced in 2021. The results of this study will serve as a valuable resource for researchers, decision-makers, academics, and tilapia entrepreneurs, enabling them to develop effective action plans to optimize tilapia production in Bangladesh and strategize for the future integration of tilapia within the country.

## Highlights

• The ARIMA (1, 1, 1) was used to predict the future trajectory of tilapia production in Bangladesh.
• The model was selected based on criteria like AIC, BIC, and analysis of ACF & PACF functions.
• The ARIMA (1, 1, 1) model forecasts a consistent upward trend in tilapia production from 2006 to 2040 in Bangladesh.
• Projected 2040 production: 690,000 MT (118% increase from 2021), range: 0.23–1.15 million MT.
• The projected production trend can guide strategic planning to address the increasing demand for tilapia in Bangladesh.

## 1 Introduction

Tilapia was introduced into Bangladesh with (*Oreochromis mossambicus* Peters*)* in 1954 and Nile tilapia (*Oreochromis niloticus)* in 1974 [1]. The local fish farmers preferred the Nile tilapia due to its robust characteristics and high disease resistance [1]. Presently tilapia has become one of the most popular aquaculture species in Bangladesh because of its adaptability to local climatic conditions, high market demand, and simple production methods [1,2]. It is now the third most important fish species in the country after “pangas” (*Pangasius hypophthalmus)* and rohu (*Labeo rohita*) and is known as the "fish for all" being a relatively cheap source of animal protein for the common people [1,2].

Aquaculture comprises a significant economic activity providing livelihood for millions of farmers and other stake holders and contributes, being the most important source of animal protein, to food security for the people of Bangladesh [[3], [4], [5]]. Along with an increase in the population that has reached more than 160 million now, the demand for fish as a primary source of protein for the people escalated substantially. This led to a steady expansion of aquaculture [3] which today accounts for approximately 57.10% of the total fish production in Bangladesh and is also a key player in the country's efforts to ensure food security for the populace [6]. In the last ten years (2011–2021), fish production from aquaculture production in Bangladesh increased by 2.34 times, and the contribution of tilapia stood at 15.11% in 2021 [2,6]. On the other hand, aquaculture provides employment opportunities for the growing population in Bangladesh [7,8].

Tilapia is an important fish species in Bangladesh with a great potential for growth due to a high demand and favorable climate for aquaculture [2,9]. Besides, tilapia is a cheap source of protein (17.1%), crude fat (0.3%), ash (1.3%), and carbohydrate (0.2%) including minerals like iron, zinc, potassium, calcium, magnesium, and sodium [10] for the people. The government of Bangladesh has been promoting aquaculture as a means of increasing fish production and supporting the country's economy [6]. However, tilapia production faces several challenges, such as high feeding and production costs, low-quality fish seed, limited processing facilities, and extreme climatic events [3,[11], [12], [13]]. These factors can make it difficult for the farmers to remain competitive in the market and gain profits [11,13]. To ensure the growth and sustainability of the tilapia production industry, Bangladesh needs to invest in the fish production sector by improving infrastructure, investing in research and technology development, and promoting sustainable aquaculture practices [11,14]. These measures can help increase fish production, improve efficiency and competitiveness, and contribute to improved food security and employment opportunities in the country [6,[14], [15], [16], [17]]. Authentic Forecasts of future production can help make effective entrepreneurial plans and optimize production processes for profitability and sustainable growth of the aquaculture industry.

The ARIMA model is a widely used statistical technique for time series data analysis and forecasting [18] which makes it an important in decision-making tool [15,[19], [20], [21], [22]]. By incorporating past values of a time series data, the ARIMA model can predict future values by making use of auto regression (AR), integration (I), and moving averages (MA). Though there are limited articles utilizing the ARIMA model for forecasting fish production, there is a noticeable absence of published literature specifically addressing the forecasting of Tilapia production in both Bangladesh and on a global scale. Therefore, this study aims to fill this gap by utilizing the ARIMA model for forecasting Tilapia production. The findings of this study can serve as a valuable resource for guiding future Tilapia production and its uses in both Bangladesh and globally.

## 2 Materials and methods

### 2.1 Data collection and processing

**Fig. 1:**
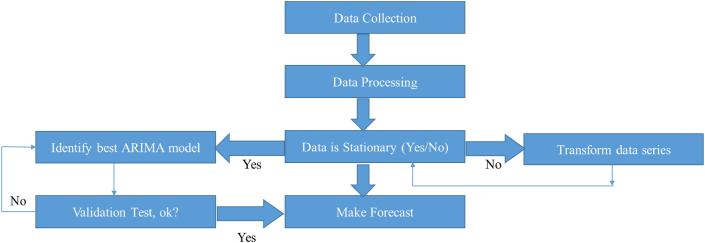
Schematic diagram of ARIMA modeling and forecasting (adopted from the Box-Jenkins methodology).

Data spanning sixteen years, covering Annual tilapia production data spanning a period of 16 years, 2006 to 2021, were collected from the Year Book of Fisheries Statistics of Bangladesh available at the official website (https://shorturl.at/gimvA) of the Department of Fisheries (DoF), Ministry of Livestock and Fisheries, Government of the People's Republic of Bangladesh. A thorough review and analysis of the data was done to identify the trends, seasonality, and outliers in the tilapia production scenario obtaining during 2006–2021 in Bangladesh. Fig. 1 shows a schematic diagram indicating various steps in ARIMA modeling and forecasting.

### 2.2 ARIMA modeling and forecasting

The present study employed ARIMA modeling and forecasting through the Box-Jenkins methodology, a widely acknowledged and significant approach for time series forecasting that relied on the utilization of time series data [23]. The ARIMA statistical model was employed to analyze and forecast time series data based on historical information spanning the last 16 years. This model was able to capture the linear associations between previous data points, current data points, and any errors or residuals within the data. The modeling process involved three components. The first component, AR(p), was used to establish a connection between past and present values, the second component, I (d), dealt with non-stationary aspects of the data and, finally, the third component, MA(q), was used to establish the relationship between the current and past errors or residuals. There were four steps in the model fitting process: identification, estimation, diagnostic checking, and forecasting. Before performing these steps, it was crucial to ensure that the tilapia production data were stationary. The past tilapia production data were not found to be normally distributed according to the Anderson-Darling test with a p-value less than the level of significance (>0.05). Non-stationarity was determined through the Augmented Dickey-Fuller (ADF) test with a p-value greater than the level of significance (>0.05). The data were transformed using the Box-Cox test to make them more stable than the original series [24,25]. The optimal value of lambda for data transformation in Box-Cox test was usually determined through rounding to 1.

#### 2.2.1 Model identification

The first step in ARIMA modeling consisted of examining the autocorrelation function (ACF) and partial autocorrelation function (PACF) of the time series data aimed to identify potential models with comparable orders. Subsequently, after identifying the possible models, estimations of the he three crucial parameters, i.e., AR(p), integration order (d), and MA(q) were carried out. The Akaike Information Criterion (AIC) and Bayesian information criterion (BIC) were employed to determine the optimal values for these parameters. The MA(q) model represents the moving average component of the ARIMA model, and is defined as:

(1) Xt=μ+εt+θ1εt‐1+θ2εt‐2+…+θqεt‐qXt=μ+εt+θ1εt‐1+θ2εt‐2+…+θqεt‐q

Here, XtXt is the observed value at time tt, μμ is the mean of the time series, εtεt is the white noise error term at time tt, and θ1,θ2, …,θqθ1,θ2, …,θq were the parameters to be estimated.

The AR(p) model represents the autoregressive component of an ARIMA model and is expressed as:

(2) Xt=φ1Xt‐1+φ2Xt‐2+…+φpXt‐p+εtXt=φ1Xt‐1+φ2Xt‐2+…+φpXt‐p+εt

Here, XtXt is the observed value at time tt, φ1,φ2, …,φpφ1,φ2, …,φp are the autoregressive parameters, and εt is the white noise error term at time t.

#### 2.2.2 Estimation of parameters

In the second stage of the ARIMA modeling process, the parameters for the chosen initial models were identified. The nonlinear least-squares method was used to estimate the parameters in the ARIMA model, as stated by a recognized previous study [26]. Multiple metrics were employed to estimate the parameters, such as root mean square error (RMSE), mean absolute percentage error (MAPE), maximum absolute percentage error (MaxAPE), mean absolute error (MAE), maximum absolute error (MaxAE), normalized Akaike Information Criterion (AIC), and normalized BIC. These metrics were considered crucial matters for assessing how well the ARIMA model aligned with the actual data and for understanding the error margin between predicted and observed values. In the calibration of the ARIMA model, the time series data were meticulously examined for stationarity, and appropriate differencing was applied as needed. Parameters, including AR(p) and MA(q), were identified using autocorrelation and partial autocorrelation functions. The model was then fitted to the training dataset, and diagnostic checks were performed on the residuals to ensure model adequacy. In the subsequent validation phase, the model was rigorously tested on unseen data, and its predictive accuracy was assessed using performance metrics such as RMSEMAE, MSE, MAPE, AIC and BIC. Iterative refinement, including adjustments to parameter values, was undertaken until a well-calibrated and validated ARIMA model was achieved, ready for reliable predictions on new observations. The model demonstrating superior forecasting ability is characterized by the smallest error criterion value [27].

(3) $$\text{MSE}=\frac{1}{n}\sum_{t=1}^{n}e_{t}^{2}$$

(4) $$\text{RMSE}=\sqrt{\frac{1}{n}\sum_{t=1}^{n}e_{t}^{2}\;}$$

(5) $$\text{MAE}=\frac{1}{n}\sum_{t=1}^{n}\left|e_{t}\right|$$

(6) $$MAPE=\frac{100\%}{n}\sum_{t=1}^{n}\left|\frac{e_{t}}{y_{t}}\right|$$

where $e^{t}$ is the error term, $y_{t}$ is the observation, and $\tilde{y_{t}}$ is the forecast, and $e_{t}^{\;}$ = $y_{t}-$ $\tilde{y_{t.}}$

(7) AIC=‐2LL+2K

where K indicates the number of estimated parameters in the model, L is the maximum value of the likelihood function for the model, and n denotes the sample size.

Bayesian Information Criteria (BIC) was introduced in 1978 as

(8) $$\text{BIC}=T^{\prime }\;\log\left(\sigma 2\right)+\left(p+q+1\right)\;{\text{logT}}^{\prime \left(\;\right)}$$

Here, σ^2^ denotes the mean square error and T′ indicates the number of observations used. The model with the lowest BIC value would be the best [28].

#### 2.2.3 Diagnostic test of residuals

The autocorrelation function (ACF) and partial autocorrelation function (PACF) of the residuals were analyzed to confirm adherence to white noise patterns. If the residuals did not show white noise, further diagnostic checks were performed to see if they were randomly and normally distributed. The distribution of lagged values in ACF and PACF did not surpass the specified threshold level, indicating a clear assessment of the accuracy of ARIMA modeling. A verification process was conducted to ensure that the estimated model accurately represented the series. The normal probability plot and histogram of residuals were examined w to assess the normal distribution of the residual dataset. These visual tools offered insights into whether the residuals conformed to a normal distribution. A close alignment of the data points with a straight line in the normal probability plot suggested that the residuals were approximately normally distributed. Additionally, the histogram displayed a bell-shaped curve, indicating a distribution consistent with normality. This analysis was integral in determining the conformity of residuals to the normal distribution assumption, with the graphical representations providing a clear indication of the distribution characteristics. The most appropriate ARIMA model was selected based on the context and specific analysis, the selection criteria being the lowest values of comparative RMSE, MAPE, MaxAPE, MAE, MaxAE, normalized AIC, and normalized BIC.

#### 2.2.4 Forecasting

The final step in ARIMA modeling was to generate forecasts and compare the accuracy of the model with other competing models using metrics such as RMSE, MAPE, MaxAPE, MAE, MaxAE, normalized AIC, and normalized BIC as the lowest values comparatively. Sample period forecasts were utilized to determine the model's confidence, while post-sample period forecasts were used to provide accurate projections for decision-making and other practical applications. The future value of a variable in the ARIMA (p, d, q) model is a linear combination of previous values and past errors represented as follows:

(9) $$\Delta^{d}+Y_{t}=\emptyset_{0}+\Delta^{d}Y_{t}=\emptyset_{0}+\emptyset_{1}\;\Delta^{d}Y_{t‐1}+\emptyset_{2}\Delta^{d}Y_{t‐2}+\ldots \ldots +\emptyset_{p}\Delta^{d}Y_{t‐p}+\varepsilon_{t}+\theta_{1}\varepsilon_{t‐1}+\theta_{2}\varepsilon_{t‐2}+\ldots ..+\theta_{q}\varepsilon_{t‐q}$$

Where, $Y_{t}$ is the actual value and ε is the random error at t, d refers to the number of differencing transformations required by the time series to get stationary. $\theta_{t}$ and $\theta_{j}$ are the coefficients, p and q are integers that are often referred to as autoregressive and moving average, respectively. The autoregressive application captured the effect of past observations, while the moving average terms accounted for the impact of past forecast errors. The combination of these components, along with the constant term (Ø0), contributed to modeling the time series data in the ARIMA framework.

### 2.3 Statistical analysis

The Microsoft Excel 2016 software was used in the organization, consolidation, and structuring of the data. Fundamental descriptive statistics of the time series data concerning tilapia production was prepared utilizing Minitab 2019. Subsequently, the results were categorized based on specific criteria. ARIMA modeling was accomplished utilizing both SPSS (version 2023) and Minitab (version 2019).

## 3 Results

Some basic descriptive statistics of tilapia production in Bangladesh from 2006 to 2021 are presented in Table 1.

**Table 1:** Basic descriptive statistics of tilapia production in Bangladesh during 2006–2021.

Indicator	Value (MT)
Minimum production	13305
Maximum production	320963
1st Quartile	38310
3rd Quartile	313527
Mean	188477
Median	240231
SE Mean	32525
Standard deviation	130099
Skewness	−0.29
Kurtosis	−1.91

**Fig. 2:**
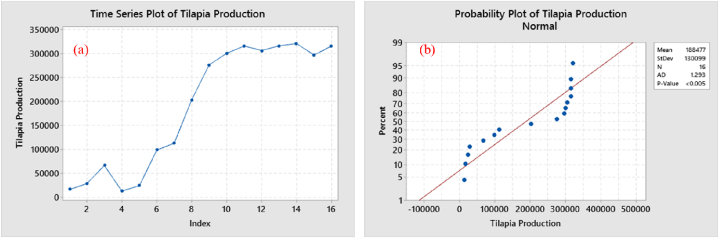
Plotting of original non-stationary data regarding tilapia production; (a) normal time series trend, (b) normality test.

**Fig. 3:**
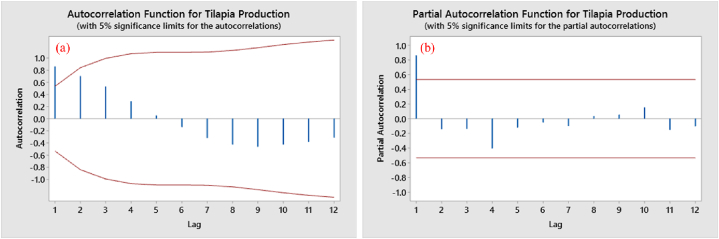
Spike distribution of original non-stationary data on tilapia production; (a) ACF, (b) PACF.

According to the Anderson-Darling test (Fig. 2b), the original data were not normally distributed as the tested value of p < 0.005 was less than the level of significance (p > 0.05). The ADF test value, p = 0.198, was greater than the level of significance (p < 0.05), so the original data were considered non-stationary (Fig. 2a). Furthermore, it can be observed that the spikes in both the ACF and PACF had crossed the threshold level in the first lag and appeared to be heading towards surpassing the threshold in other lags as well (Fig. 3a & b).

**Fig. 4:**
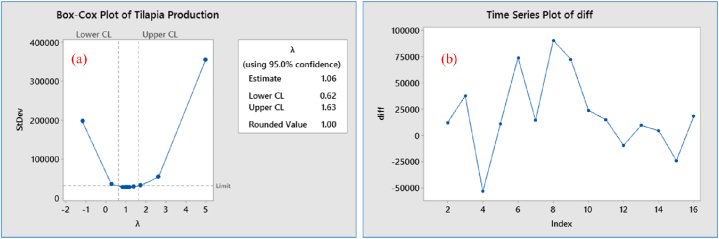
Stationary data; (a) Box-Cox plot where λ = 1, (b) time series plot of transformed data.

**Fig. 5:**
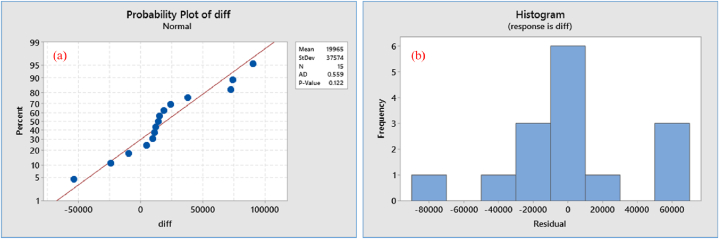
Data normality and histogram of transformed data; (a) probability plot, (b) histogram of residual.

The study revealed that the mean and variance for tilapia production in the time series data had changed, indicating that the original data series did not have a stationary pattern due to the inconsistent constancy of the mean and variance. To assess the stability of the data relative to the original series, the Box-Cox Plot test was applied. The first difference data showed a stationary pattern with a value of λ = 1 (Fig. 4a), graphical pattern of transformed data (Fig. 4b) and the p-value of the ADF test was 0.005, which was less than the significant level (p < 0.05). Since the time series production data were stationary, there were no trends or seasonal impacts, and the mean and variance of the observations remained constant over time. The normality test for the transformed data (Fig. 5a) revealed a p-value greater than the level of significance (p > 0.05) while the normal probability plot distribution (5a) and the pattern of the histogram of residual trend (Fig. 5b) indicated that the transformed data were stationary.

**Fig. 6:**
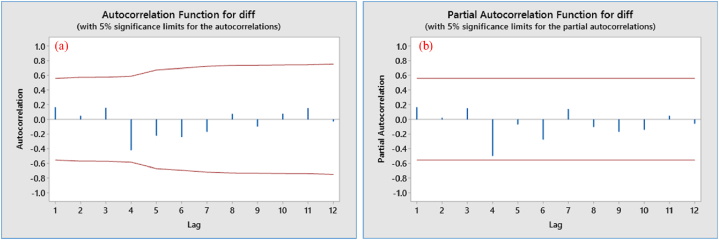
The ACF and PACF of stationary data; (a) ACF with 5% significance limit, (b) PACF with 5% significance limit.

The distribution of spikes was analyzed by examining ACF and PACF (Fig. 6a & b). These graphs represented the correlation between an observation and its lagged values in a time series data. They measured the strength and direction of the direct dependence between the observations and their lags, after accounting for the dependence with all the intermediate lags. Typically, the ACF graph showed a pattern of spikes that gradually declined as the lag increased. The spikes represented the correlation between the observation and its corresponding lag, and their magnitude indicated the strength of the correlation. Positive spikes indicated a positive correlation, meaning that an increase in the observation was associated with an increase in the lag. Negative spikes indicated a negative correlation, meaning that an increase in the observation was associated with a decrease in the lag. The ACF and PACF graphs were also accompanied by upper and lower confidence bounds, which were computed based on the properties of the underlying time series data and the number of lags. The spikes that were outside these bounds were considered statistically significant and indicated the presence of a correlation between the observation and its corresponding lag. In general, the ACF graph provided a visual representation of the dependence between the observations and their lags in a time series data, and it could be used to determine the order of the MA component in a time series model, such as ARIMA.

**Fig. 7:**
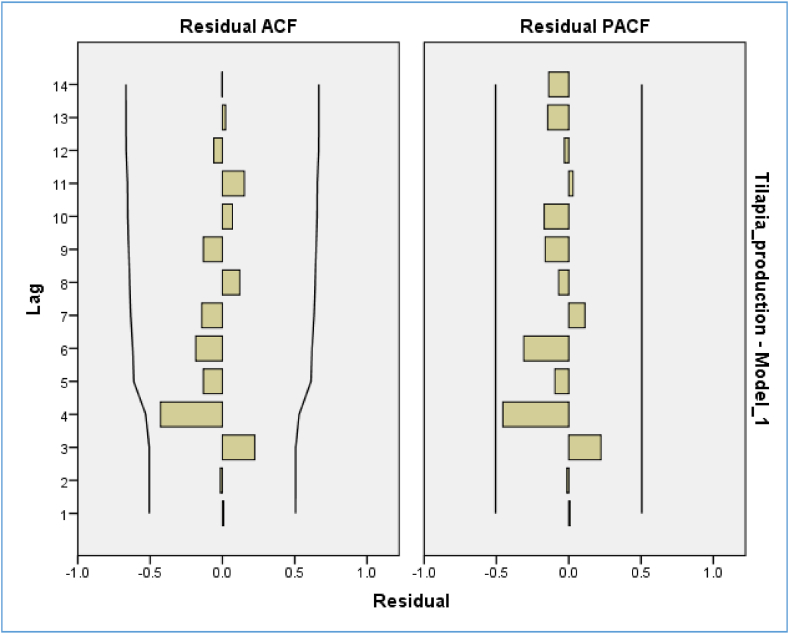
Residual ACF and PACF with normal spike distribution.

Table 2 was used to evaluate the performance of various models based on RMSE, MAPE, MaxAPE, MAE, MaxAE, and normalized BIC values. Among all the models, ARIMA (1, 1, 1) was found to have a normalized AIC and the lowest BIC values and reasonable RMSE, MAPE, MaxAPE, MAE, and MaxAE values, making it the best model for tilapia production. The residual ACF and residual PACF of the model were also analyzed, and it was found that the spikes were normally distributed. The ACF and PACF did not exceed the predefined threshold level in the distribution of lagged values. This suggests a thorough evaluation of the accuracy of ARIMA modeling (Table 2, Fig. 7). Additionally, the forecasted time series showed no evidence of white noise errors, further indicating that ARIMA (1, 1, 1) was the best fitting model (Fig. 7).

**Table 2:** Model representation with normalized BIC values. The best model is indicated with normalized AIC (*) and normalized BIC (*) value.

Model	RMSE	MAPE	MaxAPE	MAE	MaxAE	Normalized BIC	Normalized AIC
ARIMA (1,1,1)	40008.530	52.465	569.478	26284.384	75769.061	21.735*	369.56 *
ARIMA (2,1,2)	40865.857	56.686	600.803	25005.747	79936.847	22.139	383.56
ARIMA (3,1,3)	41251.798	51.617	567.538	22054.121	75510.877	22.519	421.56
ARIMA (4,1,4)	43329.596	52.075	614.533	19730.315	81763.671	22.978	1.8E+308
ARIMA (5,1,5)	53265.204	50.246	584.255	20530.748	77735.082	23.752	1.8E+308

**Fig. 8:**
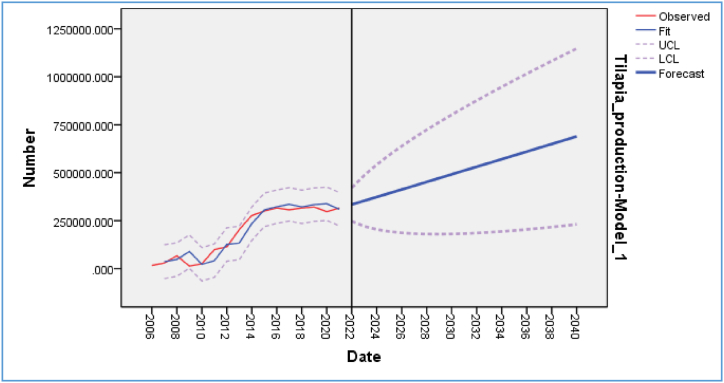
Forecast of tilapia production of Bangladesh up to the end of 2040.

The predicted values for the total tilapia production in Bangladesh for the next nineteen years (Table 3 and Fig. 8) were observed to be within the 95% confidence limits. The trend of the total tilapia production in Bangladesh is shown in Fig. 8 using the ARIMA (1, 1, 1) model. The analysis indicated a consistent increase in the total tilapia production in Bangladesh with an expected high of 690,000 MT, with an upper limit of 1.15 million MT and lower limit of 0.23 million MT by the end of 2040. This would represent a considerable increase, by 118%, over the amount recorded in 2021.

**Table 3:** Forecast of tilapia production up to the end of 2040.

Year	Tilapia production (MT) forecast	Upper confidence limit (UCL)	Lower confidence limit (LCL)
2022	334292.968	421460.174	247125.763
2023	353606.599	486540.75	220672.447
2024	373210.664	542356.962	204064.366
2025	392907.663	592492.148	193323.178
2026	412634.399	638796.451	186472.348
2027	432370.651	682358.725	182382.576
2028	452109.947	723860.264	180359.63
2029	471850.217	763749.946	179950.488
2030	491590.799	802337.698	180843.901
2031	511331.481	839846.515	182816.448
2032	531072.195	876442.603	185701.787
2033	550812.919	912253.567	189372.272
2034	570553.647	947379.833	193727.46
2035	590294.375	981902.101	198686.649
2036	610035.104	1015886.367	204183.84
2037	629775.833	1049387.422	210164.243
2038	649516.561	1082451.341	216581.781
2039	669257.29	1115117.315	223397.266
2040	688998.019	1147419.004	230577.034

## 4 Discussion

The production and demand for tilapia in Bangladesh have been steadily increasing, as reported by various sources [1,6,15]. This growth is largely due to the favorable environmental conditions, such as water depth and subtropical climate, that support the culture of tilapia [2,6]. Many studies have demonstrated that tilapia is an affordable source of animal protein, vitamins, and minerals that can be easily accessible to people [11,[29], [30], [31]]. Tilapia is the most commonly farmed fish species, with farm-raised tilapia produced in over 80 countries worldwide. The Nile tilapia (*Oreochromis niloticus*) is a particularly popular fish for farming due to its rapid growth rate in high stocking density, even in polyculture system, salt tolerance, and adaptability in wider environmental conduction [32]. It is a delicious, lean source of protein that is high in various vitamins and minerals, providing several health benefits [31]. This fish is typically farmed in rice fields and shallow water bodies, where it provides nutrients to rice plants during its growth [33,34].

The ARIMA model was considered a dynamic and accurate time series statistical model suitable for forecasting tilapia production in Bangladesh up to the year 2040. Several researchers utilized the ARIMA model in view of its value of its effectiveness in forecasts [[35], [36], [37]]. The actual data on tilapia production in the past 16 years were found to be non-stationary, so the Box-Cox Plot test was applied to transform the data into a stationary form. Among various techniques used in previous studies, the Box-Cox transformation was found to be the most efficient approach for stabilizing volatile data [24,25,38]. Notably, the data in this particular series often did not contain any noise. This study recognized that an element's influence on the accuracy of evaluations and the adequacy of findings was significant. The utilization of time series smoothing was viewed as a practical method for simplifying the process and reducing the influence of noisy elements. As a result, the data became more consistent and user-friendly. The best fit model for tilapia production in Bangladesh was identified as ARIMA (1, 1, 1) backed by lowest normalized BIC values, normalize AIC values and consistent ACF/PACF plot patterns. The lowest normalized BIC values, normalized AIC values, as well as the graphical configurations of ACF and PACF plots confirmed the model and its results. Previous several studies have documented that the models are assessed through some criteria such as the lowest normalized BIC and AIC values, the normal distribution of residual ACF and PACF spikes, along with comparatively lower values of RMSE, MAPE, MaxAPE, MAE, and MaxAE [15,18,36,37,39,40]. These findings of the present study align well with the results of several previous studies, corroborating the model's robust goodness of fit and its efficacy for forecasting [15,18,36,37,39,40]. Several previous studies emphasized the importance of using appropriate time series models to accurately describe observed data and generate reliable projections particularly for agricultural production [15,37,[41], [42], [43], [44]]. This trend looks encouraging however, this might compete with aquaculture production of other indigenous and exotic fish species. Therefore, predictive production model-based analysis of species-wise or group-wise production of tilapia as well as other fishes is also required. However, Bangladesh is a highly populated country. Therefore, tilapia can be a good item to meet the growing demand due to its high production and nutrient-rich fish. If this trend continues, it is expected, through forecasting by ARIMA, that there will be a large amount of tilapia production (690,000 MT), which is a 118% increase from the production in 2021. These findings can be beneficial for related stakeholders in achieving increased tilapia production and exploring its various uses in the future, both nationally and globally.

It is important to acknowledge the limitations involved in our attempts to model and predict tilapia production. Our emphasis was solely on statistical modeling and forecasting, which means we were not able to consider the numerous factors that impact tilapia production. Furthermore, the lack of significant influencing factors for tilapia production in the government's database over a longer period posed a challenge.

## 5 Conclusion

In this study, data from the past sixteen years on tilapia were analyzed to forecast tilapia production in Bangladesh for the next nineteen years using the ARIMA model which was found to be the appropriate model based on various statistical measures. The study suggests that with the maintenance of typical business conditions and continuation of effective strategies for aquaculture, tilapia production in the country may witness a substantial increase, of 118% by the end of 2040 from the base year of 2021 and 2040. The findings of this study provide food for thought for fisheries researchers, entrepreneurs and other stake holders, government decision-makers of Bangladesh striving to boost fish production in the country to ensure nutrition security for the populace.

## New insight

The implication of ARIMA modeling for presenting the future reality of tilapia production in Bangladesh.

## Ethical statement

This research relies on secondary source data from publicly available databases and literature (https://shorturl.at/gimvA), adhering to ethical standards in data collection, analysis, and reporting. The use of existing data follows terms of use from original sources, with proper citations. No unauthorized access or manipulation has occurred. The study respects individuals' confidentiality and privacy, aligning with ethical guidelines and promoting responsible research conduct.

## Data availability statement

The data presented in this study are available upon request from the corresponding author.

## CRediT authorship contribution statement

**Mohammad Abu Baker Siddique:** Writing – original draft, Formal analysis. **Balaram Mahalder:** Writing – review & editing, Formal analysis. **Mohammad Mahfujul Haque:** Writing – review & editing, Validation, Supervision, Conceptualization. **Mobin Hossain Shohan:** Writing – review & editing, Data curation. **Jatish Chandra Biswas:** Writing – review & editing, Funding acquisition. **Shahrina Akhtar:** Writing – review & editing, Funding acquisition. **A.K. Shakur Ahammad:** Writing – review & editing, Supervision, Conceptualization.

## Declaration of competing interest

The authors declare that they have no known competing financial interests or personal relationships that could have appeared to influence the work reported in this paper.
